# Vascular Cytokines and Atherosclerosis: Differential Serum Levels of TRAIL, IL-18, and OPG in Obstructive Coronary Artery Disease

**DOI:** 10.3390/biom14091119

**Published:** 2024-09-04

**Authors:** Katharine A. Bate, Elijah Genetzakis, Joshua Vescovi, Michael P. Gray, David S. Celermajer, Helen M. McGuire, Stuart M. Grieve, Stephen T. Vernon, Siân P. Cartland, Jean Y. Yang, Mary M. Kavurma, Gemma A. Figtree

**Affiliations:** 1 Cardiovascular Discovery Group, Kolling Institute of Medical Research, St Leonards, NSW 2065, Australia; 2 Department of Cardiology, Royal North Shore Hospital, St Leonards, NSW 2065, Australia; 3 Sydney Precision Data Science Centre, University of Sydney, Camperdown, NSW 2006, Australia; 4 School of Mathematics and Statistics, The University of Sydney, Camperdown, NSW 2006, Australia; 5 School of Medical Sciences, Faculty of Medicine and Health, University of Sydney, Camperdown, NSW 2006, Australia; 6 Heart Research Institute, The University of Sydney, Newtown, NSW 2042, Australia; 7 Department of Cardiology, Royal Price Alfred Hospital, Camperdown, NSW 2050, Australia; 8 Ramaciotti Facility for Human Systems Biology, University of Sydney, Camperdown, NSW 2006, Australia; 9 Imaging and Phenotyping Laboratory, Charles Perkins Centre, Faculty of Medicine and Health, University of Sydney, Camperdown, NSW 2006, Australia; 10 Department of Radiology, Royal Prince Alfred Hospital, Camperdown, NSW 2006, Australia; 11 The Charles Perkins Centre, The University of Sydney, Camperdown, NSW 2006, Australia

**Keywords:** cytokines, biomarkers, risk factors, atherosclerosis, coronary artery disease

## Abstract

The risk-factor-based prediction of atherosclerotic coronary artery disease (CAD) remains suboptimal, particularly in the absence of any of the standard modifiable cardiovascular risk factors (SMuRFs), making the discovery of biomarkers that correlate with atherosclerosis burden critically important. We hypothesized that cytokines and receptors associated with inflammation in CAD—tumor necrosis factor-related apoptosis-inducing ligand (TRAIL), interleukin-18 (IL-18), and osteoprotegerin (OPG)—would be independently associated with CAD. To determine this, we measured the serum biomarker levels of 993 participants from the BioHEART study who had CT coronary angiograms that were scored for severity of stenosis and plaque composition. We found that the quartiles of TRAIL, OPG, and IL-18 were significantly associated with disease scores, and that the IL-18/TRAIL and OPG/TRAIL ratios demonstrated significant differences between no CAD vs. STEMI whereas only the OPG/TRAIL ratio showed differences between no CAD and obstructive CAD (stenosis > 50%). However, these associations did not persist after adjustment for age, sex, SMuRFs, and a family history of CAD. In conclusion, TRAIL, IL-18, and OPG and the derived ratios of IL-18/TRAIL and OPG/TRAIL demonstrate significant associations with raw disease scores and risk factors, but these markers are not discriminatory biomarkers for the prediction of CAD when incorporated into multi-variable risk models.

## 1. Introduction

Atherosclerotic coronary artery disease (CAD) remains the leading cause of mortality worldwide [1] despite multi-national efforts to identify the disease early and put effective primary prevention measures in place. The increasingly recognized population of up to 20% of patients who present with heart attack without any of the Standard Modifiable cardiovascular Risk Factors (SMuRFless: no history of hypertension, hyperlipidaemia, diabetes mellitus or cigarette smoking) [2,3] contributes to the difficulty of diagnosing the disease early. The occurrence of CAD and myocardial infarction in patients in the absence of SMuRFs is becoming progressively challenging to address, and, remarkably, SMuRFless patients have higher mortality rates than those with SMuRFs [4]. The identification of blood-based biomarkers that correlate with underlying pathology in terms of disease burden or activity has the potential to significantly improve risk prediction for both the SMuRFless cohort and the wider population in general. Whilst inflammation has been known to underlie the initiation and progression of atherosclerosis [5], we have minimal clinical tools to measure this other than a C-reactive protein [5]. Even circulating levels of interleukin 1β—well known for its causal role in CAD—have not been found to be of significant diagnostic value, but many inflammatory markers remain to be assessed.

Increasing evidence suggests that the cytokines TNF-related apoptosis inducing ligand (TRAIL), osteoprotegerin (OPG), and interleukin-18 (IL-18) may interact or influence each other in advanced atherosclerotic disease. TRAIL signaling is understood to be anti-inflammatory and protective in atherosclerosis, as demonstrated by suppressed circulating levels of TRAIL in CAD [6,7,8,9,10], with preclinical studies supporting a vascular protective role [7,11,12]. We showed that myeloid-derived cells may be important contributors, as reduced TRAIL levels in CAD were concomitant with reduced *Trail* mRNA expression from patient monocytes [13]. IL-18, also shown to be elevated in acute coronary syndromes [9,14], can suppress TRAIL gene expression and contribute to the reduced TRAIL levels observed in patients with advanced CAD [7], linking TRAIL to inflammasome activation and suggesting that the ratio of IL-18 to TRAIL may be an important predictive marker. OPG is a glycoprotein TRAIL receptor involved in the inhibition of osteolysis which acts as a soluble decoy receptor for TRAIL [11]. Though the exact mechanisms behind the association between OPG and atherosclerosis remain to be clarified, elevated levels of OPG are pro-inflammatory and associated with unstable plaque development in animal models [15]. This correlates with the clinical finding that OPG levels are increased in patients with CAD [16,17] and are also associated with higher rates of ischaemic events and mortality [18,19,20]. Further research is needed to clarify the clinical significance of TRAIL, OPG, and IL-18 levels and interactions in early atherosclerotic disease.

In this study, we utilize the unique design features of the BioHEART CT and MI cohorts to unravel the potential relationship between TRAIL, OPG, and IL-18 and CAD in both acute MI and stable settings. We examine the primary hypothesis that TRAIL, OPG, and IL-18 are associated with CAD, firstly comparing these levels in acute ST-elevation myocardial infarction (STEMI) versus no STEMI, and then in association with the burden of CAD, as identified by means of computed tomography coronary angiography (CTCA), the current gold-standard non-invasive technique to definitively identify patients with and without atherosclerosis. Secondary exploratory analyses are performed to determine the association of these markers with cardiac risk factors.

## 2. Materials and Methods

### 2.1. Cohort Information

The patients included in this study were from the BioHEART biobank (Australia New Zealand Clinical Trials Registry ANZTR12618001322224, approved by the Ethics Committee of Northern Sydney Local Health District Human Research Ethics Committee: 2019/ETH08376). Written informed consent was obtained from all patients; patients were excluded if they were unwilling or unable to participate in follow-up. A total of 993 patients were included from the BioHEART-CT arm of the study, which has been described in detail previously [21]. Briefly, the study recruits adult patients who present for clinically indicated CTCA for suspected CAD. This analysis included those from the first 1000 patients recruited to BioHEART-CT who had technically adequate CTCAs, sufficient stored blood samples, and who did not have a history of coronary artery graft surgery or prior cardiac stenting (as their CTCAs could not be scored in the same manner). Clinical data were collected using a survey at the time of recruitment, and include self-reported demographic data, past medical history, medication list, smoking history, and a family history of heart disease. The SMuRFs were defined as a self-reported diagnosis for hypertension, hyperlipidaemia, diabetes mellitus, or a significant smoking history of 10 or more pack-years. In addition to the self-reported diagnosis, patients taking a statin medication were considered hyperlipidemic. Biochemical data such as fasting lipid profiles and glycated hemoglobin measurements were not available. A family history of CAD was considered significant if a first-degree relative had experienced an atherosclerotic CAD event before the age of 60 years. Patients with CAD defined on CTCA, with no features of clinical MI, were defined as having stable CAD.

Twenty patients were included from the BioHEART-MI arm of the study which recruits patients presenting for emergent angiography for STEMI. Bloods were taken at the time of primary percutaneous intervention under the emergency STEMI triage pathway. All STEMI patients had electrocardiograms (ECGs) meeting STEMI criteria and culprit plaque rupture identified on angiography. Clinical data for the STEMI cohort were collected from the medical records.

### 2.2. Biological Samples and Analysis

Blood samples were collected into serum pathology tubes (BD, Sydney, NSW, Australia) and centrifuged at 1861× *g* for 15 min at 4 °C. Serum aliquots were stored at −80 °C until analysis. Samples were thawed on ice and assayed according to manufacturer instructions using commercially available enzyme-linked immunosorbent assay (ELISA) kits. TRAIL (R&D Systems Human TRAIL/TNFSF10 Quantikine ELISA kit, #DTLR00, Minneapolis, MN, USA), OPG (R&D Systems Human Osteoprotegerin/TNFRSF11B, #DY805, Minneapolis, MN, USA), and IL-18 (Abcam Human IL-18 SimpleStep ELISA Kit, #ab215539, Melbourne, VIC, Australia) were measured. The detection ranges for TRAIL, OPG, and IL-18 were 15.6–1000 pg/mL, 6.25–4000 pg/mL, and 62.5–4000 pg/mL, respectively.

### 2.3. Imaging Acquisition and Analysis

CTCAs were obtained on 256-slice scanners using standard protocols supervised by radiologists with Royal Australia and New Zealand College of Radiologist CTCA accreditation, and radiation doses were minimized in line with current guidelines [22]. Heart rate optimization was achieved using oral metoprolol or ivabradine. Oral glyceryl trinitrate (600–800 microg) was given immediately prior to contrast injection. When heart rate control was considered optimal, prospective ECG-gating protocols were utilized for the study. If heart rate control was suboptimal, retrospective (helical) ECG-gated acquisition was performed. Reconstructions were created using appropriate software for the individual machine, and CACS was calculated using the Agatston method [23].

Image analysis was performed using the standard 17-segment model recommended by the Society of Cardiovascular Computed Tomography [24], and Gensini scores [25] were calculated for each patient. The presence of moderately obstructive CAD (stenosis > 50% in any coronary artery) and severely obstructive CAD (stenosis > 75% in any coronary artery) was also noted. To estimate the burden of soft (non-calcified) plaque present, a plaque score modifier was incorporated as follows: calcified—1; mixed—2; soft—3. This modifier was incorporated into a modification of the Gensini score, deriving a soft plaque score (SPS) via the following equation (Equation (1)):

SPS = Σ_1≤x≤17_ (stenosis score (x) × segmental significance multiplier (x) × plaque morphology multiplier (x)) − Gensini score (1)

### 2.4. Statistical Analysis

Categorical variables are presented as frequencies and percentages, and continuous variables are presented using means and standard deviations or medians with interquartile ranges, depending on the distribution of the data. TRAIL had an approximately normal distribution [26] for the purpose of parametric testing, but OPG and IL-18 were significantly right-skewed with an approximately log-normal distribution. Simple linear regression analyses were used to compare levels of TRAIL, log-transformed IL-18, and log-transformed OPG across CTCA categorical subgroups (no CAD, non-obstructive CAD, and obstructive CAD) and STEMI patients. The overall significance of the CTCA subgroups were assessed using ANOVA. Bivariate correlations of continuous data are presented as Pearson correlation coefficients for TRAIL and Spearman’s rho for OPG and IL-18, with associated 2-tailed *p*-values. Bivariate correlations of biomarker quartiles with disease scores are presented as Kendall’s tau-b correlation coefficients (τb). Correlations with associated *p* values of <0.05 were considered significantly different from zero.

Regression models were performed using the independent variables of age, sex, body mass index (BMI), hypertension, hyperlipidemia, diabetes mellitus, significant smoking history, and a family history of premature CAD—termed “risk factors”—with and without the biomarkers of interest. The association between dichotomized disease severity and biomarker concentrations was assessed using logistic regression analysis. For each biomarker, three distinct logistic regression models were constructed for the dependent variables of any CAD (Gensini > 0), moderately obstructive CAD (stenosis > 50%), and severely obstructive CAD (stenosis > 75%). The univariate and adjusted associations are presented as odds ratios with 95% confidence intervals. Linear regression was performed to assess the impact of standard risk factors on the association between biomarker and the logged non-zero values of the three disease scores as the dependent variable (logCACS, logGensini, and logSPS) for all patients. Improvement in model performance with and without the biomarker was assessed using change in R^2^ and partial F-test. The differences between the models are presented as the model R^2^, adjusted R^2^, F values, *p*-values for each model, and *p*-values for model change compared to the base model.

All data analysis was performed in SPSS Statistics (version 28.0.0.0, IBM, Armonk, NY, USA) and visualized using R Studio (version 4.3.2, Posit Software, Boston, MA, USA).

## 3. Results

### 3.1. Cohort Characteristics and Disease Burden

The demographics and risk factors for the patients included in the study are presented in Table 1. A total of 993 patients were included from the CTCA cohort, of whom 342 had no detectable coronary atherosclerosis on CTCA (Gensini score = 0). Of the remainder, 470 had non-obstructive CAD with no stenosis >50% in an epicardial artery, and 181 had obstructive atherosclerotic CAD (defined as having a plaque causing a stenosis ≥ 50% in an epicardial artery). As expected, older age (*p* < 0.001), male sex (*p* < 0.001), and an increased number of SMuRFs (*p* < 0.001) were associated with a higher burden of CAD. The 20 patients with STEMI had a similar risk factor distribution to those with obstructive CAD in the CTCA cohort. Within the CTCA cohort with detectable CAD, those with obstructive stenosis also had substantially higher quantitative measures of total disease burden vs. those with non-obstructive disease, with a median CACS of 387.5 vs. 38.2; an age- and sex-adjusted calcium percentile of 77% compared with 58%; Gensini scores of 23.5 versus 6.0; and an SPS of 20.5 compared to 4.5.

### 3.2. Relationships between Biomarkers and Disease Scores

**Figure 1:**
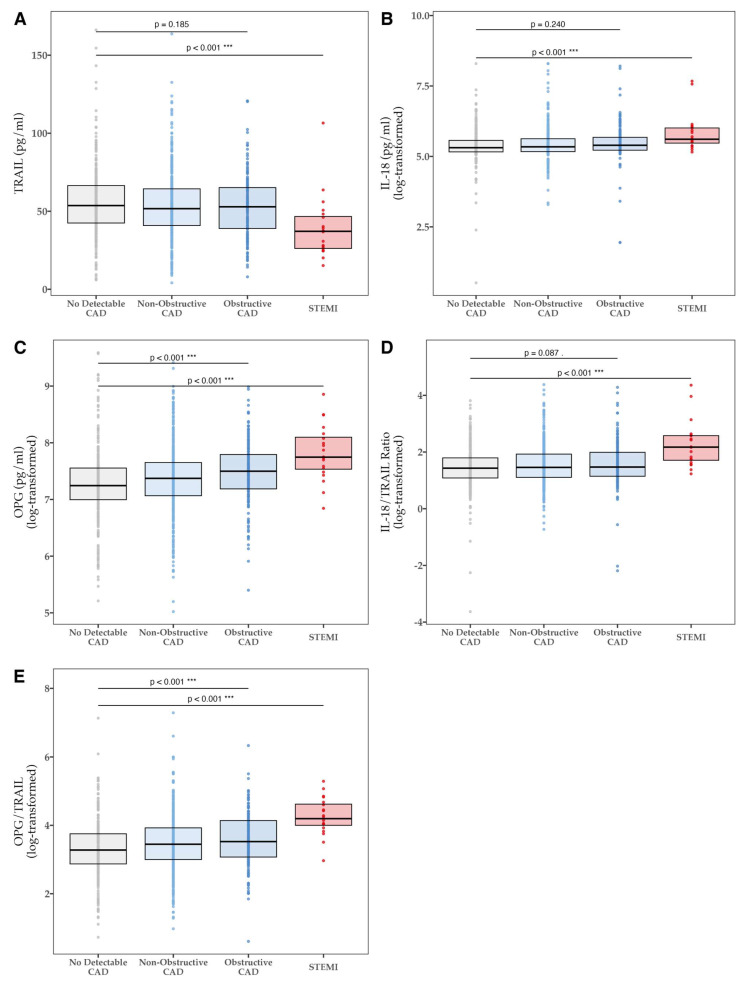
Log-transformed levels of (**A**) TRAIL, (**B**) IL-18, (**C**) OPG, (**D**) IL-18/TRAIL ratio, and (**E**) OPG/TRAIL ratio in CTCA and STEMI patients. CAD: coronary artery disease; IL-18: interleukin-18; IQR: interquartile range; OPG: osteoprotegerin; STEMI: ST-elevation myocardial infarction; TRAIL: tumor necrosis factor-related apoptosis-inducing ligand. *** *p* < 0.001. Coefficients from linear regression models are presented in Supplementary Table S1.

We first examined the levels of TRAIL, OPG, and IL-18 in STEMI and in stable CAD (Figure 1 and Table 2). The mean TRAIL concentration was ~30% lower in the STEMI cohort compared to patients with no detectable CAD (39.2 pg/mL ± 20.2 pg/mL vs. 55.8 pg/mL ± 21.8 pg/mL, *p* < 0.001). However, in a stable setting remote from MI, there was no significant difference in mean TRAIL levels between the CTCA categorical subgroups of no CAD, non-obstructive CAD, and obstructive CAD for all patients (*p* = 0.185).

In contrast, the median levels of IL-18 were higher between STEMI vs. no CAD (273.2 pg/mL ± 169.5 pg/mL vs. 202.4 pg/mL ± 87.6 pg/mL) and the log-transformed concentration was about 57% higher (*p* < 0.001). However, in the stable CTCA cohort, IL-18 levels were similar (*p* = 0.240). Additionally, the log-transformed IL-18/TRAIL ratio was significantly different between STEMI vs. no CAD (*p* < 0.001), but not within different CAD subgroups of the stable CTCA population (*p* = 0.084).

The median OPG levels were 1.7-fold higher in STEMI patients when compared to the no CAD group (2312.5 pg/mL ± 1418.9 pg/mL vs. 1396 pg/mL ± 816.5 pg/mL). The log-transformed OPG levels remained significantly higher in STEMI patients (*p* < 0.001) and showed elevated levels associated with disease severity (*p* < 0.001). An elevated OPG/TRAIL ratio is indicative of a poor prognosis in patients with MI (PMID:34313900) The median OPG/TRAIL ratio was elevated > 2-fold in STEMI patients vs. no CAD cohort (66.5 ± 46.9 vs. 26.5 ± 24.9). Similarly, the log-transformed OPG/TRAIL ratio was significantly higher in non-obstructive CAD (*p* = 0.006), obstructive CAD (*p* < 0.001), and STEMI (*p* < 0.001) versus no CAD, in a “dose-dependent” manner.

**Figure 2:**
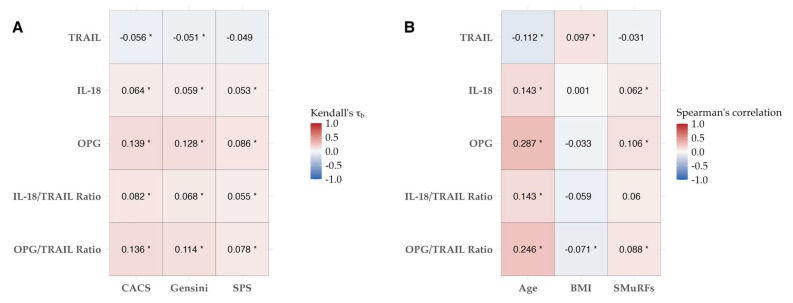
Kendall’s tau correlation (τb) heatmap for biomarker quartiles and disease score (**A**), and Spearman’s correlation (ρ) heatmap for biomarker levels with age, BMI, and the number of SMuRFs (**B**). * *p* < 0.05.

Next, biomarker scores were divided into quartiles to determine their potential directional and quantitative associations with the disease measures of CACS, Gensini, and SPS, as presented in Table 3 and Figure 2A. TRAIL demonstrated a negative correlation with quantitative measures of CAD, reaching significance for CACS (τb −0.056, *p* = 0.027) and Gensini (τb −0.049, *p* = 0.049), but not SPS. IL-18 showed a significant positive correlation with all three disease scores (CACS: τb 0.064, *p* = 0.012; Gensini: τb 0.059, *p* = 0.020; SPS: τb 0.053, *p* = 0.040). Similar findings were observed for the IL-18/TRAIL ratio (CACS: τb 0.082, *p* = 0.001; Gensini: τb 0.068, *p* = 0.009; SPS: τb 0.055, *p* = 0.039). OPG was also positively and significantly correlated with all three disease measures (CACS: τb 0.139, *p* < 0.001; Gensini: τb 0.128, *p* < 0.001; SPS: τb 0.086, *p* < 0.001), as was the OPG/TRAIL ratio (CACS: τb 0.136, *p* < 0.001; Gensini: τb 0.114, *p* < 0.001; SPS: τb 0.078, *p* = 0.003). Correlations for all biomarkers were strongest for CACS and weakest for SPS, with OPG and the OPG/TRAIL ratio demonstrating the strongest correlation with the unadjusted disease scores.

### 3.3. Relationships between Biomarkers and Cardiac Risk Factor Scores

We next explored the association between the biomarkers and the known risk factors and medications for CAD (Table 4, Supplemental Table S2). All three biomarkers and the IL-18/TRAIL and OPG/TRAIL ratios were significantly associated with age (*p* < 0.001), with moderately large correlation coefficients. BMI was positively correlated with TRAIL (r 0.097, *p* < 0.001). An increasing number of SMuRFs was positively correlated with OPG (ρ 0.106, *p* < 0.001), but was not associated with TRAIL or IL-18 alone. Participants with diabetes had significantly lower levels of TRAIL vs. those who did not have diabetes (49.71 pg/mL ± 20.76 pg/mL vs. 54.49 pg/mL ± 21.38 pg/mL, |Cohen’s d| = 0.224, *p* = 0.047), as did patients on statin medication (51.82 pg/mL ± 20.06 pg/mL vs. 55.20 pg/mL ± 21.91 pg/mL, |Cohen’s d| = 0.159, *p* = 0.019). No other clinical or demographic factors were seen to be significantly associated with the biomarkers of interest.

### 3.4. Modeling for Disease Prevalence and Severity

**Figure 3:**
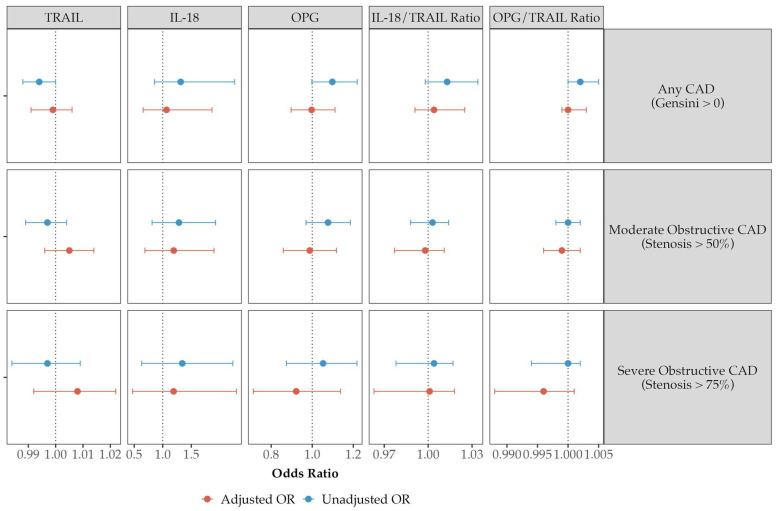
Odds ratios for the incidence of CAD from univariate and multivariable logistic regression models for TRAIL, IL-18, OPG, and the IL-18/TRAIL or OPG/TRAIL ratios.

To assess the biomarkers and derived ratios as predictors for disease prevalence, logistic regression modeling was first performed comparing the association of the biomarkers to categorical disease variables (no CAD, non-obstructive Cad, and obstructive CAD), with and without the independent variables of age, sex, BMI, hypertension, hyperlipidemia, diabetes mellitus, significant smoking history, and a family history of premature CAD. The unadjusted and adjusted odds ratios for each biomarker are presented for the prediction of any CAD (Gensini > 0), moderately obstructive CAD (stenosis > 50% in any artery), and severely obstructive CAD (stenosis > 75% in any artery) in Supplemental Table S3 and Figure 3. The odds ratios, *p*-values, and the model R^2^ values for all cardiac risk factors and biomarkers for both the total cohort and the SMuRFless sub-cohort are presented in Supplemental Table S4. Significantly higher levels of OPG were observed in the prediction of any CAD (OR 1.107 (95% CI 1.001–1.225, *p* = 0.049), but this relationship did not survive adjustment for the other cardiac risk factors. None of the other biomarkers or ratios predicted disease presence by any severity metric in the total cohort, and there were no significant associations within the SMuRFless sub-population.

Next, linear regression analysis was performed to determine whether the addition of the three biomarkers of interest impacted the strength of the model for continuous measures of disease severity. The non-zero disease scores were logged for use as the dependent variable. The relationship between the transformed disease scores and biomarkers and ratios is shown in Table 5. The standardized beta coefficients and associated *p*-values are presented for TRAIL in Supplemental Table S5, IL-18 in Supplemental Table S6, the IL-18/TRAIL ratio in Supplemental Table S7, OPG in Supplemental Table S8, and the OPG/TRAIL ratio in Supplemental Table S9. The metrics for comparison between the models are shown in Table 5, showing the model R^2^, adjusted R^2^, F values, *p*-values for each model, and the *p*-values for model change, when the biomarkers are added to the standard clinical risk factors. None of the biomarkers or ratios that were assessed contributed significantly to the prediction of disease severity for the overall cohort when incorporated into multi-variable models on top of the standard risk factors.

## 4. Discussion

In this study, we assessed the serum levels of TRAIL, IL-18, and OPG in over a thousand patients to determine associations between these markers, cardiac risk factors, and atherosclerosis burden. This is the largest study of these biomarkers in stable CAD patients performed to date, and the first to use CTCA-based measures to facilitate accurate disease scoring outside of CACS. Our results demonstrate significant differences between levels of TRAIL, OPG, and the OPG/TRAIL ratio in patients who have suffered a STEMI compared to those with stable disease, and in the stable cohort, we demonstrated differences for IL-18, OPG, and the OPG/TRAIL ratio between subgroups with increasing disease burden. When examining continuous disease scores, we showed that increasing quartiles of TRAIL had a negative correlation with CACS and Gensini score, and that increasing quartiles of IL-18, IL-18/TRAIL, OPG, and OPG/TRAIL were associated with increases in all three disease measures. However, despite these associations, the integration of the biomarkers into regression models for the prediction of disease prevalence and severity did not identify any as strong predictors of disease above and beyond standard modifiable risk factors. OPG demonstrated a weak association with the prediction of the presence of any CAD, but this relationship did not survive adjustment for standard risk factors.

Here, we identified significantly reduced CACS scores across increasing tertiles of TRAIL. Although this association did not persist in our regression data, it supports the Pittsburgh Lung Screening Study, which also showed this in ex-smokers with CAD [27]. TRAIL levels were also associated with cardiac risk factors, negatively correlating with age, and positively correlating with increasing BMI, which are findings supported by others [6,28,29,30,31]. Lower levels of TRAIL were found in patients with diabetes mellitus and in those taking a statin medication. The involvement of TRAIL in obesity and diabetes has been reviewed [32], and while there are many outstanding questions, it is clear that TRAIL plays important roles in adipogenesis [33], insulin production [14], and insulin tolerance in mouse models [34]. TRAIL’s relationship with statins is limited. In contrast to the current study, a positive correlation between the frequency of patients taking a statin and increasing quartiles of TRAIL was previously identified; however, the numbers of patients on statins were low (<5% per subgroup) and the precise levels of TRAIL in patients taking the medications were not assessed [29]. Positive correlations between total cholesterol levels and increasing TRAIL were also reported [29,30]. More studies are needed to understand this relationship.

IL-18 is a pro-inflammatory cytokine in cardiovascular disease. To our knowledge, no study has assessed IL-18 levels as a biomarker using CTCA-quantified CAD burden in patients who have not had a previous cardiac event. Here, we demonstrate a positive correlation between quartiles of IL-18 and increasing CACS scores. We also show a positive association between IL-18 levels and age. However, the direct association between Il-18 and CAD is not observed either on its own, or after inclusion of age and other standard risk factors in regression analysis. This does not exclude the possibility that IL-18 levels are reflecting coronary inflammation, given the clear role age plays in CAD. IL-18 was assessed as a biomarker for several reasons. Its expression is increased in atherosclerotic plaque [35] and in trans-coronary gradients in patients suffering acute coronary syndrome vs. controls [9], suggesting that it is released from unstable plaque directly into the coronary circulation. Polymorphic variants of the IL-18 receptor have been associated with an increased risk of MI [36]; circulating levels of IL-18 are increased in patients with unstable angina and levels were shown to correlate with the number of diseased arteries in women [37] and with increasing disease severity score [38], though no significant difference in IL-18 levels was seen between subgroups comparing CAD and non-CAD controls [36,37,38]. Following interleukin-1β inhibition in patients with prior acute coronary syndrome, a residual inflammatory risk was associated with persistently high IL-18 levels after treatment [39]. Indeed, we observed a significant increase in plasma IL-18 in our STEMI patients when compared to no CAD. IL-18 is also negatively associated with TRAIL and directly inhibits TRAIL gene expression [13]. We found that the IL-18/TRAIL ratio demonstrated significant positive associations with all three CAD scores (CACS, Gensini, and Soft Plaque Score), and the correlation of the ratio was stronger than for either IL-18 or TRAIL alone.

In clinical studies, OPG has largely been assessed epidemiologically as a measurable factor associated with adverse cardiac outcomes [12,18,40]. The OPG/TRAIL ratio is similarly associated with negative outcomes in patients following acute coronary syndrome [20] and in those with renal failure [19], but data showing an association with earlier stages of disease are lacking. In our study, we identified a dose-dependent association in unadjusted OPG and OPG/TRAIL ratios with CAD, but this finding was not observed in the regression modeling which incorporated relevant covariates. Similarly, OPG did not demonstrate an association with any of the medications used in this CAD cohort. Our data demonstrate that the unadjusted disease scores have a particularly significant association with age—a finding supported by others [41]—which could account for the subsequent associations with numbers of aggregate SMuRFs and CAD. This agrees with other data demonstrating that OPG was reported to positively correlate with measures of coronary calcification [12,42], but that OPG measurement in a cohort with suspected angina did not find that it was useful as a diagnostic marker for CAD [42].

Taken together, our data assessing levels of TRAIL, OPG, and IL-18 demonstrate significant associations with unadjusted disease metrics, consistent with our understanding of the biology. However, the lack of predictive improvement in multi-variable modeling for CAD with the addition of these biomarkers to standard risk factors suggests that the associations here are more complex. Considering that the markers were not significantly associated with the SMuRFs, this may be related to underlying biological changes that are less tangible than a concrete diagnosis of hypertension, such as metabolic dysfunction or endothelial inflammation.

The strengths of this study include the relatively large cohort size and the detailed disease scoring of the CTCAs which enabled a highly accurate determination of atherosclerosis burden. Indeed, we previously identified markers predictive for CAD using disease measures from this cohort with similar approaches [43,44,45]. However, there were several limitations to note. While CTCAs can detect epicardial atherosclerotic plaque with excellent sensitivity, microvascular disease cannot be identified on CT and may have an impact on serum levels of TRAIL, IL-18, and OPG, particularly considering their known associations with metabolic and endothelial dysfunction [15,32,46,47]. Blooming artifact from dense coronary calcification in patients with very high burdens of disease can also make the estimation of luminal stenosis inaccurate and is a technical limitation of CTCA, though only a few patients had very high calcium scores in this cohort. Additionally, CTCAs only assess the coronary vasculature, and biomarkers of atherosclerosis may be significantly altered by the presence of disease in other vascular beds. While these factors require consideration in further studies, it is reassuring that there is a significant correlation of the disease burden of coronary and peripheral vascular distributions [48] and that microvascular disease is often associated with some degree of detectable epicardial disease [49].

## 5. Conclusions

In conclusion, we have confirmed that patients with an acute STEMI have lower levels of TRAIL and higher levels of IL-18 and OPG compared with no MI patients. By using a detailed CTCA characterization of atherosclerotic disease in patients with stable CAD, we identified that levels of all three biomarkers trended significantly in the expected directions as atherosclerosis burden increased. When these biomarkers were assessed using multi-variable regression analysis adjusting for the standard factors associated with CAD, no additional predictive ability for disease prevalence or severity was demonstrated.

## Figures and Tables

**Table 1:** Cohort clinical characteristics and disease burden.

Demographics and Risk Factors	No Detectable CAD	Non-Obstructive CAD (Stenosis < 50%)	Obstructive CAD (Stenosis ≥ 50%)	STEMI	*p* Value ^#^
Number—*n*	342	470	181	20	
Age, years—mean (SD)	53 (11)	63 (10)	69 (9)	61 (10)	<0.001
Sex, male—*n* (%)	145 (42.4%)	278 (59.1%)	128 (70.7%)	10 (50.0%)	<0.001
SMuRFs—*n* (%) *					<0.001
0	113 (33.0%)	77 (16.4%)	22 (12.2%)	3 (15.0%)	
1	155 (45.4%)	199 (42.3%)	60 (33.1%)	5 (25.0%)	
2	61 (17.8%)	143 (30.4%)	67 (37.0%)	6 (30.0%)	
3	13 (3.8%)	41 (8.7%)	28 (15.5%)	4 (20.0%)	
4	0 (0.0%)	10 (2.1%)	4 (2.2%)	2 (10.0%)	
Hypertension—*n* (%)	93 (27.2%)	200 (42.6%)	93 (51.4%)	11 (55.0%)	<0.001
Hyperlipidemia—*n* (%)	162 (47.3%)	306 (65.1%)	119 (65.7%)	13 (65.0%)	<0.001
Diabetes Mellitus—*n* (%)	21 (6.1%)	43 (9.1%)	22 (12.2%)	6 (30.0%)	0.003
Significant Smoking History—*n* (%)	40 (11.7%)	99 (21.1%)	60 (33.1%)	7 (35.0%)	<0.001
Significant Family History of Premature CAD—*n* (%)	69 (20.2%)	98 (20.9%)	37 (20.4%)	6 (30.0%)	0.7
Atrial Fibrillation—*n* (%)	31 (9.1%)	65 (13.8%)	24 (13.3%)	0 (0%)	0.057
Previous TIA/Stroke—*n* (%)	12 (3.5%)	34 (7.2%)	5 (2.8%)	0 (0%)	0.035
Peripheral Arterial Disease—*n* (%)	3 (0.9%)	5 (1.1%)	6 (3.3%)	0 (0%)	0.14
Anti-Platelet Medication—*n* (%)	46 (13.5%)	85 (18.1%)	45 (24.9%)	5 (25.0%)	0.009
Anti-Coagulant Medication—*n* (%)	20 (5.8%)	50 (10.6%)	18 (9.9%)	1 (5.0%)	0.085
Statin—*n* (%)	62 (18.1%)	184 (39.1%)	84 (46.4%)	5 (25.0%)	<0.001
Beta Blocker—*n* (%)	34 (9.9%)	68 (14.5%)	38 (21.0%)	5 (25.0%)	0.003
ACE Inhibitor/ARB—*n* (%)	68 (19.9%)	169 (36.0%)	79 (43.6%)	9 (45.0%)	<0.001
CACS—median (IQR)	0 (0)	38.2 (143)	387.5 (807.6)	N/A	<0.001
Calcium Percentile—median (IQR)	0 (0)	58 (44)	77 (34)	N/A	<0.001
Gensini Score—median (IQR)	0 (0)	6.0 (7.5)	23.5 (17.0)	N/A	<0.001
Soft Plaque Score—median (IQR)	0 (0)	4.5 (7.5)	20.5 (16.0)	N/A	<0.001

ACE: angiotensin-converting enzyme; ARB: angiotensin receptor blocker; CACS: coronary artery calcium score; CAD: coronary artery disease; IQR: interquartile range; N/A: not applicable; TIA: transient ischemic attack; SD: standard deviation; SMuRF: standard modifiable cardiovascular risk factor; STEMI: ST-elevation myocardial infarction. * Sum of the number of SMuRFs (major risk factors) per patient. **^#^** Kruskal–Wallis rank sum test; Pearson’s Chi-squared test; Fisher’s exact test.

**Table 2:** Biomarker levels for CAD subgroups.

Serum Biomarker Levels	No Detectable CAD	Non-Obstructive CAD (Stenosis < 50%)	Obstructive CAD (Stenosis ≥ 50%)	STEMI	*p* Value ^#^
TRAIL [pg/mL]—mean (SD)					0.005
All	55.78 (21.88)	53.29 (21.34)	52.90 (20.31)	39.24 (20.19)	
SMuRFless	55.77 (23.99)	53.51 (21.07)	56.14 (26.93)		
IL-18 [pg/mL]—median (IQR)					0.004
All	202.4 (174.4–262.0)	208.7 (175.9–278.0)	220.1 (185.2–292.4)	273.2 (238.3–407.8)	
SMuRFless	204.7 (174.6–278.5)	196.5 (171.2–252.0)	206.1 (173.0–275.9)		
IL-18/TRAIL ratio—median (IQR)					<0.001
All	4.18 (2.96–6.01)	4.30 (3.02–6.87)	4.35 (3.14–7.32)	8.79 (5.53–13.18)	
SMuRFless	4.34 (3.05–6.67)	3.81 (2.96–6.53)	4.03 (2.81–7.43)		
OPG [pg/mL]—median (IQR)					<0.001
All	1396.9 (1091.7–1908.2)	1591.2 (1171.4–2104.0)	1805.1 (1321.6–2422.8)	2312.5 (1871.3–3290.2)	
SMuRFless	1294.6 (948.2–1974.4)	1462.9 (1017.3–2051.6)	1709.6 (1257.4–2695.2)		
OPG/TRAIL ratio—median (IQR)					<0.001
All	26.5 (17.7–42.6)	31.4 (20.1–50.7)	33.9 (21.6–62.8)	66.5 (54.7–101.6)	
SMuRFless	24.8 (14.8–42.6)	29.6 (16.8–50.1)	35.1 (19.8–85.7)		

CAD: coronary artery disease; IL-18: interleukin-18; IQR: interquartile range; OPG: osteoprotegerin; STEMI: ST-elevation myocardial infarction; TRAIL: tumor necrosis factor-related apoptosis-inducing ligand. **^#^** ANOVA F-test in total cohort.

**Table 3:** Correlations between quartiles of TRAIL, IL-18, OPG, and the ratios of IL-18/TRAIL and OPG/TRAIL and disease scores.

Biomarker Associations with Disease—Unadjusted	Quartile 1 (Lowest)	Quartile 2	Quartile 3	Quartile 4 (Highest)	Correlation Coefficient (τb)	*p*-Value
TRAIL [pg/mL]–median (IQR)						
CACS	28.6 (0.0, 203.9)	7.1 (0.0, 98.1)	4.5 (0.0, 162.5)	5.2 (0.0, 118.0)	−0.056	0.027
Gensini Score	5.8 (0.0, 17.0)	3.5 (0.0, 9.5)	3.5 (0.0, 12.5)	3.5 (0.0, 10.5)	−0.049	0.049
Soft Plaque Score	2.5 (0.0, 11.6)	2.5 (0.0, 8.0)	1.5 (0.0, 9.1)	1.5 (0.0, 8.6)	−0.046	0.069
IL-18 [pg/mL]–median (IQR)						
CACS	2.7 (0.0, 115.4)	6.8 (0.0, 125.5)	9.6 (0.0, 138.8)	28.8 (0.0, 195.6)	0.064	0.012
Gensini Score	2.5 (0.0, 9.5)	4.0 (0.0, 11.3)	3.5 (0.0, 11.5)	5.0 (0.0, 13.0)	0.059	0.020
Soft Plaque Score	0.0 (0.0, 8.0)	2.5 (0.0, 10.0)	2.3 (0.0, 8.5)	3.0 (0.0, 10.1)	0.053	0.040
IL-18/TRAIL ratio–median (IQR)						
CACS	3.0 (0.0, 112.1)	4.6 (0.0, 122.5)	7.0 (0.0, 131.4)	36.3 (0.0, 227.6)	0.082	0.001
Gensini Score	2.5 (0.0, 10.0)	3.5 (0.0, 10.6)	2.8 (0.0, 11.0)	6.0 (0.0, 14.8)	0.068	0.009
Soft Plaque Score	0.0 (0.0, 8.5)	3.0 (0.0, 9.0)	0.0 (0.0, 8.1)	3.5 (0.0, 10.8)	0.055	0.039
OPG [pg/mL]–median (IQR)						
CACS	0.1 (0.0, 77.4)	1.5 (0.0, 91.2)	20.7 (0.0, 194.5)	32.6 (0.0, 237.6)	0.139	<0.001
Gensini Score	2.5 (0.0, 9.5)	2.5 (0.0, 9.1)	5.0 (0.0, 13.0)	6.0 (0.0, 17.0)	0.128	<0.001
Soft Plaque Score	0.0 (0.0, 9.0)	0.0 (0.0, 6.6)	3.0 (0.0, 10.0)	3.3 (0.0, 12.0)	0.086	<0.001
OPG/TRAIL ratio—median (IQR)						
CACS	0.1 (0.0, 78.0)	4.2 (0.0, 113.1)	18.4 (0.0, 163.0)	32.9 (0.0, 271.4)	0.136	<0.001
Gensini Score	2.5 (0.0, 10.0)	3.5 (0.0, 10.0)	3.5 (0.0, 11.5)	6.5 (0.0, 17.3)	0.114	<0.001
Soft Plaque Score	0.0 (0.0, 9.0)	0.0 (0.0, 8.0)	2.5 (0.0, 9.0)	3.5 (0.0, 11.8)	0.078	0.003

CACS: coronary artery calcium score; IL-18: interleukin-18; IQR: interquartile range; OPG: osteoprotegerin; τb: Kendall’s tau coefficient; TRAIL: tumor necrosis factor-related apoptosis-inducing ligand.

**Table 4:** Correlation coefficients between TRAIL, IL-18, OPG, and the IL-18/TRAIL and OPG/TRAIL ratios with continuous variables, and effect size of the difference between groups for categorical cardiac risk factors.

		TRAIL		IL-18		IL-18/TRAIL		OPG		OPG/TRAIL	
Continuous Variables	*n*	Spearman’s rho (ρ)	*p*-value	Spearman’s rho (ρ)	*p*-value	Spearman’s rho (ρ)	*p*-value	Spearman’s rho (ρ)	*p*-value	Spearman’s rho (ρ)	*p*-value
Age (years)	993	−0.112	<0.001	0.143	<0.001	0.143	<0.001	0.287	<0.001	0.246	<0.001
BMI (kg/m^2^)	990	0.097	0.002	0.001	0.830	−0.059	0.044	−0.033	0.323	−0.071	0.023
SMuRFs	993	−0.031	0.331	0.062	0.041	0.060	0.055	0.106	<0.001	0.088	0.005
Categorical Variables	*n*	|Cohen’s d|	*p*-value	|Cohen’s d|	*p*-value	|Cohen’s d|	*p*-value	|Cohen’s d|	*p*-value	|Cohen’s d|	*p*-value
Male	551	0.039	0.545	0.024	0.735	0.037	0.616	0.030	0.634	0.008	0.914
Hypertension	386	0.024	0.707	0.030	0.632	0.034	0.587	0.121	0.070	0.041	0.532
Hyper- lipidaemia	587	0.006	0.920	0.033	0.611	0.030	0.631	0.017	0.797	0.032	0.660
Diabetes Mellitus	86	0.224	0.047	0.174	0.451	0.122	0.288	0.143	0.176	0.074	0.277
Significant Smoking	199	0.113	0.153	0.042	0.387	0.004	0.948	0.025	0.746	0.003	0.971
Significant FH of CAD	204	0.059	0.450	0.042	0.650	0.030	0.696	0.092	0.175	0.076	0.575
TIA or Stroke	51	0.079	0.584	0.388	0.432	0.472	0.428	0.080	0.505	0.022	0.784
PAD	14	0.040	0.880	0.221	0.573	0.296	0.467	0.016	0.960	0.004	0.982
Inflammatory Arthritis	105	0.005	0.963	0.007	0.886	0.021	0.746	0.017	0.842	0.049	0.485
Atrial Fibrillation	120	0.005	0.962	0.098	0.061	0.074	0.218	0.112	0.314	0.007	0.911
Statin	330	0.159	0.019	0.013	0.877	0.073	0.435	0.061	0.343	0.052	0.426
Anti- Coagulant	89	0.029	0.795	0.175	0.523	0.229	0.490	0.027	0.761	0.033	0.599
Anti-Platelet	176	0.149	0.074	0.053	0.0.293	0.027	0.645	0.026	0.728	0.008	0.898
Beta Blocker	140	0.009	0.917	0.060	0.619	0.014	0.854	0.085	0.241	0.041	0.674
ACE Inhibitor or ARB	316	0.091	0.180	0.096	0.308	0.121	0.257	0.089	0.190	0.105	0.175

ACE: angiotensin-converting enzyme; ARB: angiotensin receptor blocker; BMI: body mass index; FH: family history; IL-18: interleukin-18; OPG: osteoprotegerin; PAD: peripheral arterial disease; SMuRF: standard modifiable cardiovascular risk factor; TIA: transient ischemic attack; TRAIL: tumor necrosis factor-related apoptosis-inducing ligand; (ρ) Spearman’s rank correlation coefficient.

**Table 5:** Comparison of multi-variable linear regression models predicting disease severity, demonstrating the model change with the incorporation of biomarker combinations for each disease score, when added to standard risk factors.

Comparison of Linear Regression Models	R^2^	Adjusted R^2^	F Value	*p* Value for Model	*p* Value for Model Change
Log(Gensini)	Total Cohort				
Risk Factors	0.217	0.208	22.21	<0.001	
+TRAIL	0.217	0.206	19.72	<0.001	0.790
+IL-18	0.218	0.207	19.75	<0.001	0.621
+IL18/TRAIL	0.217	0.206	19.71	<0.001	0.985
+OPG	0.217	0.206	19.71	<0.001	0.962
+OPG/TRAIL	0.218	0.206	19.74	<0.001	0.686
Log(CACS)	Total Cohort				
Risk Factors	0.247	0.236	23.64	<0.001	
+TRAIL	0.247	0.235	20.98	<0.001	0.895
+IL-18	0.249	0.237	21.23	<0.001	0.192
+IL18/TRAIL	0.247	0.235	21.03	<0.001	0.564
+OPG	0.247	0.235	20.98	<0.001	0.897
+OPG/TRAIL	0.248	0.236	21.14	<0.001	0.302
Log(SPS)	Total Cohort				
Risk Factors	0.093	0.079	6.75	<0.001	
+TRAIL	0.095	0.080	6.13	<0.001	0.291
+IL-18	0.093	0.078	6.01	<0.001	0.729
+IL18/TRAIL	0.093	0.078	5.99	<0.001	0.961
+OPG	0.100	0.084	6.45	<0.001	0.054
+OPG/TRAIL	0.095	0.080	6.14	<0.001	0.272

## Data Availability

The data that support this study are available from the corresponding author upon reasonable request.

## Supplementary Materials

The following supporting information can be downloaded at: https://www.mdpi.com/article/10.3390/biom14091119/s1, Table S1: Marginal estimates from univariate linear regressions for biomarkers (dependent variables) against disease severity. Table S2: Differences in serum levels of TRAIL, OPG and IL-18 in patients with and without categorical cardiac risk factors and prescribed medications. Table S3: Comparison of univariate logistic regression odds ratios (biomarker only) to multi-variable logistic regression with adjustment for the risk factors: biomarker plus age, sex, BMI, hypertension, hyperlipidaemia, diabetes mellitus, significant smoking history and family history of premature coronary disease. Top panel showing results for all patients, bottom panel showing results for the SMuRFless sub-cohort. Table S4: Odds ratios from multi-variable logistic regression modelling for categorical disease metrics (dependent variables) including all risk factors and biomarkers listed below (independent variables). Top panel showing results for all patients, bottom panel showing results for the SMuRFless sub-cohort. Table S5: Standardized beta coefficients and P-values from multi-variable linear regression models including TRAIL, showing all patients (top) and SMuRFless patients (bottom) for logged non-zero values of all three disease scores. Table S6: Standardized beta coefficients and P-values from multi-variable linear regression models including IL-18, showing all patients (top) and SMuRFless patients (bottom) for logged non-zero values of all three disease scores. Table S7: Standardized beta coefficients and *p*-values from multi-variable linear regression models including the ratio of IL-18/TRAIL, showing all patients (top) and SMuRFless patients (bottom) for logged non-zero values of all three disease scores. Table S8: Standardized beta coefficients and *p*-values from multi-variable linear regression models including OPG, showing all patients (top) and SMuRFless patients (bottom) for logged non-zero values of all three disease scores. Table S9: Standardized beta coefficients and *p*-values from multi-variable linear regression models including the OPG/TRAIL ratio, showing all patients (top) and SMuRFless patients (bottom) for logged non-zero values of all three disease scores.
