# Supplementary material for: Vascular Cytokines and Atherosclerosis: Differential Serum Levels of TRAIL, IL-18, and OPG in Obstructive Coronary Artery Disease
Source: Biomolecules. 2024 Sep 4;14(9):1119. doi: 10.3390/biom14091119 (PMC11430378; doi:10.3390/biom14091119)
Supplement: Supplementary file 1 [file biomolecules-14-01119-s001.zip › biomolecules-3068940-supplementary.pdf]

Supplemental Materials

Supplemental Table S1……………….. Page 2

Supplemental Table S2……………….. Page 3

Supplemental Table S3………………..Page 4

Supplemental Table S4………………..Page 5

Supplemental Table S5………………..Page 6

Supplemental Table S6………………..Page 6

Supplemental Table S7………………..Page 7

Supplemental Table S8………………..Page 7

Supplemental Table S9………………..Page 8

Supplemental Table S1. Marginal estimates from univariate linear regressions for biomarkers (dependent variables) against disease severity.

Logistic Regression	Trail		Log IL-18		Log OPG	
	Marginal Estimate (95% CI)	*P* Value	Marginal Estimate (95% CI)	*P* Value	Marginal Estimate (95% CI)	*P* Value
No CAD (Reference)	55.78 (-)	-	5.39 (-)	-	7.26 (-)	-
Non-Obstructive CAD (Stenosis ≤ 50%)	53.29 (50.32-56.27)	0.100	5.43 (5.35-5.51)	0.335	7.36 (7.21-7.45)	0.013
Obstructive CAD (Stenosis > 50%)	52.90 (49.05-56.75)	0.141	5.48 (5.37-5.58)	0.096	7.47 (7.30-7.58)	<0.001
STEMI	39.24 (29.61-48.87)	<0.001	5.84 (5.58-6.10)	<0.001	7.82 (7.55-8.09)	<0.001

Logistic Regression	Log IL-18/TRAIL		Log IL-18/OPG	
	Marginal Estimate (95% CI)	*P* Value	Marginal Estimate (95% CI)	*P* Value
Non CAD (Reference)	1.45 (-)	-	3.32 (-)	-
Non-Obstructive CAD (Stenosis ≤ 50%)	1.54 (1.44-1.65)	0.084	3.48 (3.28-3.68)	0.006
Obstructive CAD (Stenosis > 50%)	1.59 (1.46-1.72)	0.045	3.58 (3.35-3.81)	<0.001
STEMI	2.27 (1.94-2.60)	<0.001	4.25 (4.04-4.46)	<0.001

Supplemental Table S2. Differences in serum levels of TRAIL, OPG and IL-18 in patients with and without categorical cardiac risk factors and prescribed medications.

**Biomarker Associations with Risk Factors and Medications**	**TRAIL**				**OPG**			**IL-18**		
	N	Mean	SD	*P*-value	Median	IQR	*P*-value	Median	IQR	*P*-value
Male	551	54.45	21.17	0.545	1502.9	954.2	0.623	207.6	96.1	0.731
Female	442	53.62	21.61		1623.6	884.1		211.1	103.3	
Hypertension (-)	607	54.28	22.23	0.707	1476.6	863.7	0.062	207.0	97.0	0.658
Hypertension (+)	386	53.76	19.9		1642.1	1055.6		214.8	104.0	
Hyperlipidaemia (-)	406	54.16	22.01	0.920	1517.4	991.4	0.789	206.5	97.6	0.589
Hyperlipidaemia (+)	587	54.02	20.91		1568.6	883.0		210.5	100.8	
Diabetes Mellitus (-)	907	54.49	21.38	**0.047**	1527.5	871.5	0.187	208.4	99.8	0.453
Diabetes Mellitus (+)	86	49.71	20.76		1819.2	937.9		221.7	116.9	
Significant Smoking History (-)	794	54.56	21.51	0.153	1556.8	913.5	0.732	208.4	99.4	0.402
Significant Smoking History (+)	199	52.15	20.69		1553.6	1015.3		212.0	108.1	
Significant Family History (-)	789	53.82	21.71	0.450	1562.0	951.8	0.174	208.8	97.2	0.651
Significant Family History (+)	204	55.09	19.96		1527.5	848.3		208.0	113.6	
TIA or Stroke (-)	942	54.17	21.31	0.584	1548.2	907.7	0.496	208.3	99.5	0.445
TIA or Stroke (+)	51	52.48	22.40		1701.9	1127.9		226.3	105.2	
Peripheral Arterial Disease (-)	979	54.07	21.38	0.880	1557.6	923.5	0.959	208.8	99.4	0.552
Peripheral Arterial Disease (+)	14	54.93	20.45		1300.8	770.6		188.1	121.5	
Inflammatory Arthritis (-)	888	54.07	21.67	0.963	1553.0	907.9	0.849	208.0	98.1	0.891
Inflammatory Arthritis (+)	105	55.17	18.62		1568.6	1141.3		223.0	124.5	
Atrial Fibrillation (-)	873	54.07	21.58	0.962	1547.2	886.9	0.314	208.8	99.6	0.064
Atrial Fibrillation (+)	120	54.17	19.80		1640.0	1241.4		202.7	100.1	
Statin (-)	663	55.20	21.91	**0.019**	1490.7	895.5	0.379	204.3	93.8	0.870
Statin (+)	330	51.82	20.06		1670.4	1000.1		220.8	106.2	
Anti-Coagulant (-)	904	54.06	21.52	0.930	1537.1	894.9	0.785	208.0	98.4	0.512
Anti-Coagulant (+)	89	54.27	25.35		1767.4	1158.8		218.9	109.1	
Anti-Platelet (-)	817	54.64	21.91	0.074	1517.8	920.1	0.753	208.4	98.9	0.298
Anti-Platelet (+)	176	51.47	18.42		1633.3	865.6		215.6	109.7	
Beta Blocker (-)	853	54.05	21.43	0.917	1553.6	899.7	0.262	208.7	99.8	0.645
Beta Blocker (+)	140	54.25	21.00		1557.2	1020.6		210.4	98.7	
ACE Inhibitor or ARB (-)	667	54.70	21.99	0.180	1521.2	874.3	0.184	205.8	95.1	0.311
ACE Inhibitor or ARB (-)	316	52.75	19.91		1625.5	1013.9		217.0	105.2	

Supplemental Table S3. Comparison of univariate logistic regression odds ratios (biomarker only) to multi-variable logistic regression with adjustment for the risk factors: biomarker plus age, sex, BMI, hypertension, hyperlipidaemia, diabetes mellitus, significant smoking history and family history of premature coronary disease. Top panel showing results for all patients, bottom panel showing results for the SMuRFless sub-cohort.

**Logistic Regression**	**Any CAD (Gensini > 0, n = 650)**		**Moderate Obstructive CAD (Stenosis > 50%, n = 181)**		**Severe Obstructive CAD (Stenosis > 75%, n = 56)**	
**All Patients (n=993)**	Odds Ratio (95% CI)	*P* Value	Odds Ratio (95% CI)	*P* Value	Odds Ratio (95% CI)	*P* Value
TRAIL Unadjusted	0.994 (0.988-1.000)	0.069	0.997 (0.989-1.004)	0.411	0.997 (0.984-1.009)	0.620
TRAIL + Risk Factors	0.999 (0.991-1.006)	0.762	1.005 (0.996-1.014)	0.327	1.008 (0.992-1.022)	0.308
IL18 Unadjusted	1.314 (0.855-2.260)	0.479	1.284 (0.813-1.925)	0.957	1.342 (0.631-2.228)	0.847
IL18 + Risk Factors	1.066 (0.656-1.864)	0.629	1.192 (0.687-1.898)	0.968	1.190 (0.472-2.292)	0.863
IL18/TRAIL Unadjusted	1.013 (0.998-1.034)	0.190	1.003 (0.988-1.014)	0.654	1.004 (0.978-1.017)	0.621
IL18/TRAIL + Risk Factors	1.004 (0.991-1.025)	0.633	0.998 (0.977-1.011)	0.857	1.001 (0.963-1.018)	0.973
OPG Unadjusted	1.098 (0.998-1.219)	0.066	1.077 (0.970-1.186)	0.133	1.053 (0.874-1.218)	0.518
OPG + Risk Factors	0.997 (0.897-1.111)	0.969	0.988 (0.859-1.118)	0.866	0.922 (0.713-1.138)	0.500
OPG/TRAIL Unadjusted	1.002 (1.000-1.005)	0.117	1.000 (0.998-1.002)	0.549	1.000 (0.994-1.002)	0.961
OPG/TRAIL + Risk Factors	1.000 (0.999-1.003)	0.587	0.999 (0.996-1.002)	0.599	0.996 (0.988-1.001)	0.259
**SMuRFless Patients (n=211)**	**Any CAD (Gensini > 0, n = 99)**		**Moderate Obstructive CAD (Stenosis > 50%, n = 22)**		**Severe Obstructive CAD (Stenosis > 75%, n = 8)**	
TRAIL Unadjusted	0.997 (0.985-1.009)	0.600	1.002 (0.984-1.021)	0.805	1.017 (0.992-1.042)	0.184
TRAIL + Risk Factors	1.003 (0.989-1.017)	0.698	1.010 (0.989-1.032)	0.352	1.021 (0.992-1.052)	0.157
IL18 Unadjusted	0.843 (0.316-2.250)	0.734	2.544 (0.828-7.819)	0.103	0.402 (0.003-59.708)	0.721
IL18 + Risk Factors	0.646 (0.236-1.771)	0.396	2.156 (0.624-7.443)	0.224	0.242 (0.001-102.361)	0.242
IL18/TRAIL Unadjusted	1.006 (0.968-1.006)	0.761	1.031 (0.987-1.077)	0.166	0.931 (0.749-1.156)	0.516
IL18/TRAIL + Risk Factors	0.992 (0.952-1.034)	0.713	1.025 (0.978-1.075)	0.305	0.911 (0.711-1.168)	0.463
OPG Unadjusted	1.137 (0.950-1.361)	0.162	1.106 (0.874-1.399)	0.403	0.927 (0.549-1.565)	0.777
OPG + Risk Factors	1.012 (0.822-1.245)	0.910	0.974 (0.729-1.303)	0.860	0.716 (0.362-1.419)	0.339
OPG/TRAIL Unadjusted	1.005 (0.998-1.013)	0.167	1.000 (0.997-1.004)	0.872	0.996 (0.975-1.018)	0.735
OPG/TRAIL + Risk Factors	1.002 (0.996-1.007)	0.542	0.999 (0.993-1.006)	0.853	0.987 (0.960-1.015)	0.367

Supplemental Table S4. Odds ratios from multi-variable logistic regression modelling for categorical disease metrics (dependent variables) including all risk factors and biomarkers listed below (independent variables). Top panel showing results for all patients, bottom panel showing results for the SMuRFless sub-cohort.

**Logistic Regression**	**Any CAD (Gensini > 0, n = 650)**		**Moderate Obstructive CAD (Stenosis > 50%, n = 181)**		**Severe Obstructive CAD (Stenosis > 75%, n = 56)**	
**All Patients (n=993)**	Odds Ratio (95% CI)	*P* Value	Odds Ratio (95% CI)	*P* Value	Odds Ratio (95% CI)	*P* Value
Age	1.126 (1.105-1.146)	<0.001	1.098 (1.076-1.119)	<0.001	1.114 (1.077-1.153)	<0.001
Sex	5.314 (3.705-7.621)	<0.001	3.227 (2.182-4.771)	<0.001	8.473 (3.652-19.658)	<0.001
Body Mass Index	1.019 (0.986-1.053)	0.257	0.999 (0.961-1.040)	0.978	1.009 (0.943-1.080)	0.786
Hypertension	1.319 (0.926-1.877)	0.125	1.335 (0.918-1.943)	0.131	0.912 (0.487-1.707)	0.773
Hyperlipidaemia	1.508 (1.088-2.090)	0.014	0.999 (0.682-1.463)	0.997	0.805 (0.430-1.504)	0.496
Diabetes Mellitus	1.803 (0.923-3.520)	0.084	1.345 (0.733-2.466)	0.338	1.801 (0.750-4.323)	0.188
Significant Smoking History	1.639 (1.052-2.554)	0.029	1.846 (1.229-2.773)	0.003	2.104 (1.118-3.960)	0.021
Significant Family History	1.619 (1.086-2.414)	0.018	1.572 (1.000-2.472)	0.050	2.548 (1.236-5.251)	0.011
TRAIL	0.999 (0.991-1.006)	0.762	1.004 (0.996-1.013)	0.330	1.008 (0.992-1.023)	0.345
IL18	1.000 (1.000-1.000)	0.991	1.000 (1.000-1.000)	0.953	1.000 (1.000-1.001)	0.848
OPG	1.000 (1.000-1.000)	0.612	1.000 (1.000-1.000)	0.910	1.000 (1.000-1.000)	0.563
All Patients: Model R^2	0.415		0.257		0.266	
**SMuRFless Patients (n=211)**	**Any CAD (Gensini > 0, n = 99)**		**Moderate Obstructive CAD (Stenosis > 50%, n = 22)**		**Severe Obstructive CAD (Stenosis > 75%, n = 8)**	
Age	1.105 (1.071-1.140)	<0.001	1.097 (1.049-1.147)	<0.001	1.084 (1.010-1.164)	0.025
Sex	4.219 (2.070-8.600)	<0.001	3.502 (1.134-10.811)	0.029	9.155 (0.947-88.504)	0.056
Body Mass Index	0.994 (0.932-1.060)	0.856	1.088 (0.974-1.216)	0.134	1.076 (0.904-1.280)	0.410
Significant Family History	1.460 (0.683-3.123)	0.329	0.742 (0.218-2.517)	0.632	4.587 (0.952-22.092)	0.058
TRAIL	1.003 (0.989-1.017)	0.712	1.011 (0.989-1.033)	0.340	1.021 (0.990-1.053)	0.187
IL18	1.000 (0.999-1.001)	0.402	1.001 (1.000-1.002)	0.219	0.999 (0.993-1.004)	0.645
OPG	1.000 (1.000-1.000)	0.938	1.000 (1.000-1.000)	0.925	1.000 (0.999-1.000)	0.383
SMuRFless: Model R^2	0.356		0.267		0.246	

Supplemental Table S5. Standardized beta coefficients and *P*-values from multi-variable linear regression models including TRAIL, showing all patients (top) and SMuRFless patients (bottom) for logged non-zero values of all three disease scores.

**Linear Regression - TRAIL**	**Log(Gensini)**		**Log(CACS)**		**Log(SPS)**	
**All Patients**	Standardized Beta	*P* Value	Standardized Beta	*P* Value	Standardized Beta	*P* Value
Age	0.401	<0.001	0.437	<0.001	0.241	<0.001
Sex	0.265	<0.001	0.273	<0.001	0.176	<0.001
Body Mass Index	-0.001	0.971	0.022	0.557	-0.001	0.981
Hypertension	0.077	0.037	0.078	0.043	0.065	0.136
Hyperlipidaemia	0.049	0.169	0.079	0.035	0.001	0.979
Diabetes Mellitus	0.071	0.047	0.064	0.083	0.069	0.106
Significant Smoking History	0.050	0.167	0.016	0.660	0.071	0.098
Significant Family History	0.046	0.204	0.019	0.609	0.088	0.040
TRAIL	0.010	0.790	0.005	0.895	0.045	0.291
**SMuRFless Patients**						
Age	0.319	0.002	0.336	0.002	0.276	0.021
Sex	0.168	0.089	0.318	0.003	0.052	0.662
Body Mass Index	0.160	0.109	0.246	0.021	0.163	0.168
Significant Family History	-0.118	0.223	-0.073	0.485	0.007	0.948
TRAIL	-0.066	0.507	-0.014	0.898	0.027	0.823

Supplemental Table S6. Standardized beta coefficients and *P*-values from multi-variable linear regression models including IL-18, showing all patients (top) and SMuRFless patients (bottom) for logged non-zero values of all three disease scores.

**Linear Regression – IL18**	**Log(Gensini)**		**Log(CACS)**		**Log(SPS)**	
**All Patients**	Standardized Beta	*P* Value	Standardized Beta	*P* Value	Standardized Beta	*P* Value
Age	0.400	<0.001	0.438	<0.001	0.236	<0.001
Sex	0.265	<0.001	0.274	<0.001	0.176	<0.001
Body Mass Index	0.000	1.000	0.024	0.532	0.005	0.915
Hypertension	0.078	0.035	0.079	0.039	0.066	0.131
Hyperlipidaemia	0.050	0.167	0.079	0.033	0.003	0.949
Diabetes Mellitus	0.069	0.053	0.061	0.098	0.064	0.134
Significant Smoking History	0.049	0.169	0.017	0.643	0.069	0.108
Significant Family History	0.046	0.201	0.020	0.586	0.089	0.039
IL18	0.017	0.621	0.047	0.192	0.014	0.729
**SMuRFless Patients**						
Age	0.317	0.001	0.333	0.002	0.258	0.025
Sex	0.167	0.086	0.315	0.003	0.029	0.796
Body Mass Index	0.149	0.125	0.245	0.020	0.165	0.146
Significant Family History	-0.111	0.245	-0.069	0.507	0.020	0.855
IL18	0.127	0.188	0.054	0.605	0.201	0.072

Supplemental Table S7. Standardized beta coefficients and *P*-values from multi-variable linear regression models including the ratio of IL-18/TRAIL, showing all patients (top) and SMuRFless patients (bottom) for logged non-zero values of all three disease scores.

**Linear Regression – IL-18/TRAIL**	**Log(Gensini)**		**Log(CACS)**		**Log(SPS)**	
**All Patients**	Standardized Beta	*P* Value	Standardized Beta	*P* Value	Standardized Beta	*P* Value
Age	0.399	<0.001	0.436	<0.001	0.235	<0.001
Sex	0.265	<0.001	0.273	<0.001	0.176	<0.001
Body Mass Index	0.000	0.995	0.024	0.534	0.005	0.917
Hypertension	0.078	0.036	0.079	0.039	0.066	0.133
Hyperlipidaemia	0.050	0.166	0.079	0.034	0.003	0.946
Diabetes Mellitus	0.070	0.049	0.063	0.088	0.065	0.128
Significant Smoking History	0.049	0.172	0.016	0.660	0.068	0.110
Significant Family History	0.046	0.205	0.019	0.609	0.088	0.040
IL18/TRAIL	0.001	0.985	0.021	0.564	0.002	0.961
**SMuRFless Patients**						
Age	0.318	0.001	0.336	0.002	0.255	0.027
Sex	0.160	0.100	0.316	0.003	0.022	0.850
Body Mass Index	0.159	0.104	0.246	0.020	0.179	0.115
Significant Family History	-0.125	0.191	-0.074	0.476	-0.006	0.960
IL18/TRAIL	0.141	0.144	0.032	0.757	0.185	0.100

Supplemental Table S8. Standardized beta coefficients and *P*-values from multi-variable linear regression models including OPG, showing all patients (top) and SMuRFless patients (bottom) for logged non-zero values of all three disease scores.

**Linear Regression - OPG**	**Log(Gensini)**		**Log(CACS)**		**Log(SPS)**	
**All Patients**	Standardized Beta	*P* Value	Standardized Beta	*P* Value	Standardized Beta	*P* Value
Age	0.399	<0.001	0.436	<0.001	0.249	<0.001
Sex	0.265	<0.001	0.273	<0.001	0.173	<0.001
Body Mass Index	0.000	0.994	0.023	0.547	0.006	0.886
Hypertension	0.078	0.036	0.078	0.041	0.067	0.123
Hyperlipidaemia	0.050	0.166	0.079	0.034	-0.001	0.976
Diabetes Mellitus	0.070	0.049	0.064	0.085	0.067	0.114
Significant Smoking History	0.049	0.172	0.016	0.661	0.063	0.141
Significant Family History	0.046	0.204	0.019	0.609	0.084	0.048
OPG	0.002	0.962	0.005	.0897	-0.082	0.054
**SMuRFless Patients**						
Age	0.308	0.003	0.344	0.002	0.291	0.014
Sex	0.159	0.112	0.326	0.003	0.067	0.565
Body Mass Index	0.141	0.152	0.248	0.020	0.185	0.110
Significant Family History	-0.129	0.185	-0.069	0.509	0.015	0.894
OPG	0.092	0.364	-0.026	0.813	-0.121	0.295

Supplemental Table S9. Standardized beta coefficients and *P*-values from multi-variable linear regression models including the OPG/TRAIL ratio, showing all patients (top) and SMuRFless patients (bottom) for logged non-zero values of all three disease scores.

**Linear Regression - OPG/TRAIL**	**Log(Gensini)**		**Log(CACS)**		**Log(SPS)**	
**All Patients**	Standardized Beta	*P* Value	Standardized Beta	*P* Value	Standardized Beta	*P* Value
Age	0.401	<0.001	0.440	<0.001	0.240	<0.001
Sex	0.266	<0.001	0.274	<0.001	0.177	<0.001
Body Mass Index	-0.001	0.984	0.022	0.561	0.003	0.941
Hypertension	0.077	0.037	0.076	0.048	0.065	0.139
Hyperlipidaemia	0.049	0.172	0.077	0.040	0.000	0.998
Diabetes Mellitus	0.071	0.047	0.065	0.079	0.066	0.119
Significant Smoking History	0.049	00.172	0.015	0.676	0.066	0.122
Significant Family History	0.046	.201	0.020	0.589	0.090	0.036
OPG/TRAIL	-0.014	0.686	-0.038	0.302	-0.046	0.272
**SMuRFless Patients**						
Age	0.328	0.001	0.338	0.002	0.271	0.021
Sex	0.170	0.084	0.322	0.003	0.046	0.694
Body Mass Index	0.150	0.126	0.245	0.020	0.169	0.143
Significant Family History	-0.129	0.188	-0.068	0.519	0.006	0.957
OPG/TRAIL	0.069	0.482	-0.017	0.869	0.003	0.981
